# Partial Ureteropelvic Junction Disruption after Blunt Trauma: Case Report

**DOI:** 10.1155/2011/541705

**Published:** 2011-10-05

**Authors:** Jalal Eddine El Ammari, Youness Ahallal, Mohammed Jamal El Fassi, M. Hassan Farih

**Affiliations:** Department of Urology, University Hospital Center Hassan II, Fes, Morocco

## Abstract

Partial ureteropelvic junction disruption as a result of blunt trauma is rare and frequently missed by the initial trauma evaluation. Delays in diagnosis have also been associated with significant morbidity. A high index of suspicion should lead to appropriate investigations, and the management will be determined by the severity of the disruption. We present herein a 24-year-old man who was admitted to the Emergency Room with multiple organ injuries caused by a severe blunt trauma. Emergency celiotomy was performed for massive hemoperitoneum and shattered spleen which led to splenectomy. The diagnosis of partial UPJ disruption was missed preoperatively and suspected in CT scan after appearance of flank tender mass. Confirmation was obtained in retrograde ureteropyelography and treated conservatively with indwelling ureteral stent. We present herein an extensive review of the literature to examine the current status of this entity and to determine if improvements could be made in the diagnosis and treatment.

## 1. Introduction

Ureteral injuries from external violence are not common. Most injuries reported involved the ureteropelvic junction. The diagnosis is usually late in a large proportion of cases. A high index of suspicion should determine the appropriate evaluation, and the management options depend on the severity of the disruption. We report a case of partial UPJ disruption secondary to blunt trauma that was treated successfully with indwelling ureteral stent.

## 2. Case Presentation

A 24-year-old male Moroccan patient presented to the emergency department with multiple injuries secondary to a high-velocity motor vehicle collisions: multiple rib fractures, left pneumothorax, and splenic laceration. As the patient was hemodynamically unstable, the patient was taken immediately to the operating room. An urgent abdominal ultrasonography showed massive hemoperitoneum and shattered spleen. Chest radiography showed multiple left rib fractures (6th–10th) with hemothorax. An urgent splenectomy was therefore performed. A chest tube drained 500 mL of blood. No hematuria was noted at placement of the Folley catheter. The patient was transferred to the surgical intensive care unit for observation. Her hemodynamic parameters and hemoglobin were maintained stable over the following 3 days. 

**Figure 1:**
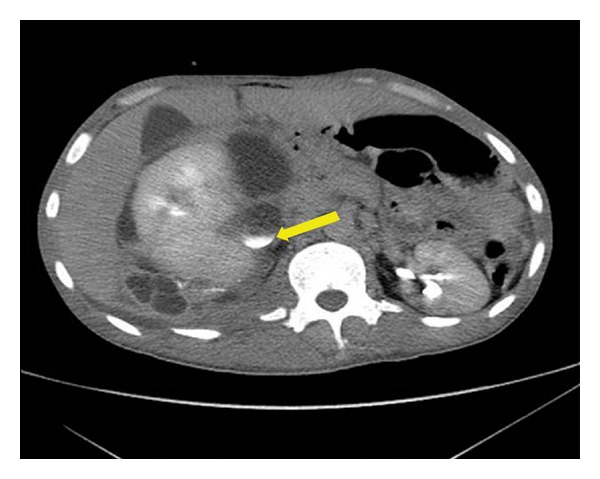
CT scan showing urinoma, contrast extravasation (arrow) with a right pelvicaliceal dilatation.

**Figure 2:**
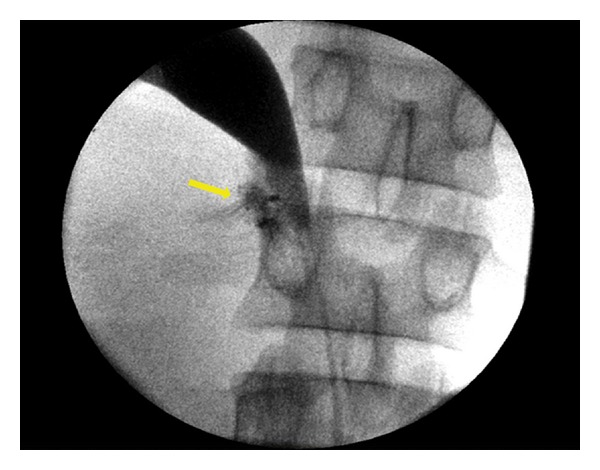
Retrograde ureteropyelogram showing opacification of all the upper urinary tract with extravasation of the contrast material in the ureteropelvic junction (arrow).

Ten days later, the patient complained of low back and right flank pain. The abdomen was swollen in the physical examination, and a subsequent CT scan revealed urinoma, contrast extravasation (Figure 1) and a right pelvicaliceal dilatation without ureteral opacification. A retrograde ureteropyelogram revealed a partial ureteropelvic junction injury (Figure 2), and a double-J stent was successfully inserted. The urinoma was drained percutaneously. 6 weeks after the double-J stent removal, the patient was fine and did not show any evidence of stricture.

## 3. Discussion

Ureteral trauma is rare; it represents less than 1% of all urological injuries. Partial ureteropelvic junction disruption secondary to blunt trauma is even exceptional [1, 2]. Most cases are complete avulsion and are most likely associated with multiple organ injuries. Most reported cases are children (adults represent only around 30% of the reported cases). The right side is involved 3 times more often than the left side [3].

 As ureters are well protected anatomically by the psoas muscles and bony pelvis, ureteral injury from external violence is extremely rare [1]. The blunt ureteropelvic junction injury occurs through two successive events: first, stretching of the body and then, sudden deceleration causing ureteral compression against a transverse process or the last rib [4]. 

Ureteropelvic junction disruption continues to be diagnosed late in a large proportion of cases. A delay of 36 hours or longer occurs from 0% to 57% of patients [4, 5]. Missing ureteropelvic junction injuries led to increased morbidity including upper tract obstruction, urinoma, urinary fistula formation, sepsis, nephrectomy rate (32% in delayed recognition versus 4, 5% if treated immediately), and longer hospital stay [1, 6].

Several reasons were evoked to explain this diagnosis delay. One of which could be explained by investigations focused on the genitourinary tract (IVP, CT scanning with delayed images) was being performed based on initial clinical parameters. However, no set of initial clinical parameters (hematuria, direct flank tender mass, ecchymosis, fracture of a transverse process of lumbar vertebra, blunt abdominal trauma, or deceleration injury) have been found to reliably predict ureteropelvic junction or ureteral injuries [2, 7–9]. The second reason is that most delays occur secondary to hemodynamic instability and underlying injuries, both of which are common with trauma severe enough to result in blunt ureteral injury [10].

Efforts have been made to define indications for imaging the genitourinary tract after blunt trauma [11, 12]. Using the criteria of Nicolaisen et al. either gross hematuria or microhematuria and shock are insufficient since only 53% of all adult cases of ureteropelvic junction disruptions reported in the literature had either of these at presentation. By adding associated injuries, such as spinal or pelvic fractures or flank ecchymosis and tenderness, to the criteria for imaging as suggested by Hardeman et al. more cases would be detected [13]. Reviewing the literature using the criteria of Hardeman et a1.would lead to 96% of cases being imaged. It had been assumed that if a patient was imaged then the correct diagnosis would be made most of the time.

For the physicians involved in trauma management, a critical factor leading to an accurate diagnosis of ureteropelvic junction disruption is having a high index of suspicion in situations of high velocity blunt trauma, especially those involving a rapid deceleration injury [1]. When this type of accident occurs, imaging of the genitourinary tract is indicated for microhematuria with shock, gross hematuria, direct flank tenderness, ecchymosis, or multisystem trauma. In such cases, if the initial contrast-enhanced spiral CT reveals minimal abnormalities of the kidneys, delayed films done 5 to 8 minutes after the injection of contrast material would be useful to exclude more accurately an ureteropelvic junction disruption [2, 14]. This finding is particularly true when there are subtle abnormalities, such as perinephric fluid collections, hematomas, or failure to visualize the ureter despite enhancement of the kidney [4].

Medina et al. reported that both pre- and intraoperative diagnostic testing is inaccurate and direct visualization of the ureter is necessary to make the diagnosis. However they found that all of missed injuries of their series occurred in the upper portion of the ureter in presence of a retroperitoneal hematoma though exploration of the upper ureter in the face of a large hematoma may be more difficult or incomplete, especially if there is concern about a concomitant renal injury [7]. Our patient did not show any sign leading to retroperitoneal organ lesion (no hematuria nor retroperitoneal hematoma) that is why we did not explore the retroperitoneum. 

The European Association of Urology recommends in the case of hemodynamic instability when immediate laparotomy is undertaken, a “one-shot intravenous urogram” on the operating table is recommended 10 minutes after intravenous injection of contrast medium. Intravenous urography is not routinely used in the context of blunt trauma. However, it can be performed on low risk patients following surgical intervention to assess ureteral potency. If ureteropelvic junction lesion in a stable patient is still suspected, retrograde ureterorenography makes the definitive diagnosis. Treatment using ureteral stent could be performed in the same procedure [12].

 The American Association for the Surgery of Trauma (AAST) has classified ureteral injuries in 5 grades (Table 1). Partial injuries can be defined as grade I and II lesions corresponding to hematoma and laceration lower than 50% of circumference, respectively [12].

The management strategy of ureteral trauma dependents on the type of ureteral injury, should be considered for the management the timing of diagnosis, associated injuries, and degree of injury.

Blunt partial ureteropelvic junction disruption can be managed conservatively with urine diversion using indwelling ureteral stent or percutaneous nephrostomy [1, 4, 12, 15]. To date, no relevant clinical trial compared outcomes between these techniques; however, most authors consider ureteral stenting to be the best treatment option as it provides canalization and stabilization of the ureter and offers secure drainage of the kidney. If the injury is encountered during immediate surgical exploration, primary closure of the ureteral ends over a stent may be recommended. Certain surgical principles should be respected. These include adequate debridement of damaged tissue, careful mobilization of ureter or kidney to assure tension-free anastomosis, watertight spatulated closure, isolation from associated injuries, and external nonsuction drainage of the area of ureteral repair [12]. To achieve this, the use of ureteral stent with proximal urinary diversion in delayed treatment would be suitable. The stenting should be kept in place for at least 3 weeks. The patient should have follow-up intravenous pyelography after 3 to 6 months or sooner if the patient reports clinical symptoms. If there is evidence of stricture, a variety of endourological or open surgical techniques could be indicated [1, 12].

## 4. Conclusion

Partial UPJ disruptions are rare. The diagnosis continues to be late in a large proportion of cases. A high index of suspicion in situations of high velocity blunt trauma, especially those involving a rapid deceleration injury, should determine the appropriate evaluation, and the management options depend on the severity of the disruption. Delayed scanning of the upper urinary tract after conventional portovenous-phase imaging and retrograde uretero-pyelography were useful to obtain definitive diagnosis. The partial ureteropelvic junction disruption can be successfully managed with indwelling ureteral stent placement offering an alternative approach to open surgery.

## Figures and Tables

**Table 1:** AAST organ injury severity scale for the ureter [15].

Grade	Description of injury
I	Haematoma only
II	Laceration <50% of circumference
III	Laceration >50% of circumference
IV	Complete tear <2 cm of devascularization
V	Complete tear >2 cm of devascularization
